# Exploring the Interactome of the Queuine Salvage Protein DUF2419 in *Entamoeba histolytica*

**DOI:** 10.3390/cells13221900

**Published:** 2024-11-18

**Authors:** Jun Ye, Meirav Trebicz-Geffen, Serge Ankri

**Affiliations:** Department of Molecular Microbiology, Ruth and Bruce Rappaport Faculty of Medicine, Technion-Israel Institute of Technology, Haifa 3525433, Israel; junye@campus.technion.ac.il (J.Y.); meiravg@technion.ac.il (M.T.-G.)

**Keywords:** DUF2419, *Entamoeba histolytica*, interactome, queuosine, tRNA, proteasome

## Abstract

*Entamoeba histolytica* causes amebiasis, a significant global health issue, with millions affected annually, especially in developing countries. EhDUF2419, an important protein involved in *E. histolytica*’s queuine salvage pathway and its interaction network, remains unclear. To explore this, we transfected *E. histolytica* trophozoites with a plasmid encoding Myc-tagged EhDUF2419 and achieved successful overexpression. Through immunoprecipitation with the Myc antibody followed by mass spectrometry, we identified 335 proteins interacting with Myc-tagged EhDUF2419, including over 100 ribosomal proteins, along with translation initiation and elongation factors, and aminoacyl-tRNA synthetases. Ribosome purification revealed the presence of EhDUF2419 in ribosomal protein-enriched fractions. Treatment with queuosine (Q) significantly reduced the EhDUF2419 protein levels and decreased the Q-modified tRNA in Myc-tagged EhDUF2419 overexpressing trophozoites. This effect, which was Q-dependent, was not observed in strains carrying an empty vector control or overexpressing a truncated form of EhDUF2419 lacking catalytic activity. The reduction in the EhDUF2419 protein levels was regulated by proteasome-mediated degradation, as evidenced by the reduced degradation in the presence of MG132, a proteasome inhibitor. Our study uncovers the novel interaction of EhDUF2419 with ribosomal proteins and its regulation by the proteasome machinery, providing new insights into its role in *E. histolytica* and potential therapeutic strategies.

## 1. Introduction

Amebiasis, caused by the protozoan parasite *Entamoeba histolytica*, is a significant parasitic infection. The World Health Organization estimates that each year, approximately 50 million people in regions such as India, Southeast Asia, Africa, and Latin America contract amebic dysentery and amebiasis, leading to at least 100,000 deaths. Transmission typically occurs through the ingestion of contaminated food or water. Once ingested, the cysts excyst in the intestinal lumen, where the trophozoites then colonize the large intestine. The life cycle continues as both trophozoites and cysts are excreted in stools. While most infected individuals (90%) remain asymptomatic, symptomatic cases can experience diarrhea, abdominal pain, and fever. Severe infections can lead to life-threatening abscesses in the liver or other organs [1]. Treatment generally involves antibiotics, with metronidazole being the standard therapy for invasive amebiasis in both adults and children [2]. However, metronidazole can have side effects such as nausea, headaches, metallic taste, and even neurotoxicity. Additionally, there are emerging concerns about *E. histolytica* developing resistance to metronidazole, highlighting the need for alternative treatments [3,4].

The gut microbiota, consisting of numerous bacteria, coexists with *E. histolytica* in the intestinal environment. *E. histolytica* not only consumes these bacteria as a food source but also interacts intricately with them. One key interaction involves the salvage of queuine from gut bacteria by *E. histolytica*. Queuine, the nucleobase of queuosine (Q), is highly conserved across bacteria, plants, fishes, insects, and mammals. While bacteria can synthesize queuine de novo, eukaryotes lack this capability and must acquire queuine either through diet or from their intestinal microbiota [5,6,7]. Q is a modified nucleoside found in the first position of transfer RNA (tRNA) anticodons with G_34_U_35_N_36_, such as Asp, Asn, His, and Tyr [8]. The enzyme responsible for incorporating Q into tRNA instead of G34 is tRNA guanine transglycosylase (TGT), which in *E. histolytica* is a heterodimer composed of EhQTRT1 and EhQTRTD1 [9]. Once incorporated into the corresponding tRNA, queuine regulates the translational speed and fidelity in eukaryotes [10,11] and influences the expression of genes involved in oxidative stress responses and virulence in *E. histolytica* [9]. The two known transporter families that salvage Q precursors are QPTR/COG1738 [12] and QrtT/QueT [13] in *Escherichia coli*. A recent study identified three new families that facilitate Q precursor (preQ0 and preQ1) transport: a ureide permease (PF07168) from *Acidobacteriota* bacterium, a hemolysin III family protein (PF03006) from *Bifidobacterium breve*, and a major facilitator superfamily protein (PF07690) from *Bartonella henselae* [14]. DUF2419 catalyzes the formation of Q from queuine in *Schizosaccharomyces pombe* [15] and in *E. histolytica* [16]. Based on structural predictions, the similarity between DUF2419 and 8-oxoguanine DNA glycosylases implies a potential hydrolase function [15,17,18]. Despite its known role in queuine salvage, the interaction network of EhDUF2419 (EHI_098190) in *E. histolytica* remain poorly understood. In this study, we employed immunoprecipitation (IP) combined with mass spectrometry (MS) to investigate the EhDUF2419 interactome, uncovering its multifaceted role in the parasite’s cellular processes.

## 2. Materials and Methods

### 2.1. E. histolytica Culture

The HM-1:IMSS strain of *E. histolytica* was kindly supplied by Prof. Samudrala Gourinath from Jawaharlal Nehru University in New Delhi, India. The amoebae were cultivated at 37 °C in 13 × 100 mm screw-capped Pyrex glass tubes using Diamond’s TYI-S-33 medium until they reached the exponential growth phase. The trophozoites were then collected from the culture tubes by tapping them and subsequently centrifuging, following previously established protocols [19].

### 2.2. Construction of the Myc-Tagged EhDUF2419 (MycEhDUF2419) or Truncated EhDUF2419 Vector (MycTrunEhDUF2419)

To construct the Myc-tagged EhDUF2419 expression vector, the EhDUF2419 gene was amplified from *E. histolytica* cDNA using sense and antisense primers (Table 1) incorporating SmaI and XhoI sites, respectively. The resulting PCR product was cloned into the pGEM-T Easy Vector System (Promega, Beit Haemek, Israel), digested with SmaI and XhoI, and subsequently subcloned into the pKT-3M expression vector (kindly supplied by Prof. Upinder Singh from the Stanford University School of Medicine, Stanford, CA, USA; [20]) that included a Myc-tag and had been previously linearized with SmaI and XhoI. The plasmids were sequenced to verify the absence of unwanted mutations.

For the MycTrunEhDUF2419, we identified a putative active site based on the DUF2419 protein from *Sphaerobacter thermophilus* (StDUF2419), as described in a referenced article [18]. The sequence alignment showed that EhDUF2419 and StDUF2419 share 33% identity, with the active site residues D298 and W302 in StDUF2419 corresponding to D280 and W284 in EhDUF2419. We truncated EhDUF2419 starting just before site D280, added a stop codon and restriction sites, and amplified this segment from *E. histolytica* cDNA using specific primers (Table 1). The remaining steps were identical to those used for constructing the Myc-tagged EhDUF2419 vector.

### 2.3. Transfection of E. histolytica Trophozoites

The transfection of the *E. histolytica* trophozoites was carried out following the method outlined by Olvera et al. [21].

### 2.4. Quantitative Real-Time PCR (qRT-PCR)

The total RNA was isolated from either control or Myc-tagged EhDUF2419 trophozoites using TRI reagent (Merck, Rehovot, Israel), and its concentration was determined using a nanodrop spectrophotometer (Thermo Fisher Scientific (Heysham), Lancashire, UK). Reverse transcription was carried out using the RevertAid First Strand cDNA Synthesis Kit (Thermo Fisher Scientific (Heysham), Lancashire, UK) following the manufacturer’s instructions. The primers utilized for amplifying EhDUF2419 and rDNA are detailed in Table 1. The qRT-PCR was conducted using the qPCR-Bio SyGreen Mix Hi-ROX (PCR Biosystems, London, UK) as per the manufacturer’s protocol and run on the Real-Time PCR QuantStudio3 (Thermo Fisher Scientific (Heysham), Lancashire, UK) with the following cycling conditions: initial denaturation at 95 °C for 2 min, followed by 40 cycles of denaturation at 95 °C for 5 s, and annealing/extension at 50 °C for 30 s. The melting curve analysis was performed under the following conditions: 95 °C for 15 s, 60 °C for 1 min, and a final step at 95 °C for 15 s. The relative fold change was determined using the 2^−∆∆Ct^ method [22]. The qRT-PCR values were normalized to the expression level of the rDNA gene. PCR amplification controls were included for each primer pair to confirm product formation.

### 2.5. Western Blot Analysis

Western blotting was conducted on the total protein extracts from the *E. histolytica* trophozoites (50 μg) following a previously established protocol [9]. For the experiments involving Q (a gift from Prof. Peter C. Dedon, MIT, USA) treatment, the trophozoites were incubated with 0.1 μM Q for 2 days prior to protein extraction. In the experiments involving MG132 (Merck, Rehovot, Israel) treatment, the trophozoites were exposed to 20 μM MG132 for 2 days. For the combined treatment conditions, the trophozoites were co-treated with 20 μM MG132 and 0.1 μM Q for 2 days. The proteins were separated on a 12% SDS gel and then transferred to a nitrocellulose membrane (Whatman, Protran BA83, Merck, Rehovot, Israel). The membranes were subsequently blocked with 5% skim milk and incubated with mouse anti-Myc antibody (Cell Signaling Technology, Danvers, MA, USA) (diluted 1:1000) overnight at 4 °C. Following incubation, the blots were washed and probed with a secondary antibody (Jackson ImmunoResearch, West Grove, PA, USA) at room temperature for 1 h, followed by detection using an enhanced chemiluminescence reagent (WesternBright^TM^ ECL, Advansta, CA, USA). They were then photographed with Fusion FX7 Edge Spectra.

### 2.6. Immunoprecipitation

We followed a protocol similar to that outlined in reference [23], with slight adjustments. First, 5 × 10^6^ cells were lysed in 3 mL of lysis buffer containing 20 mM Tris-HCl (pH 7.5), 1 mM MgCl_2_, 10% (vol/vol) glycerol, 50 mM NaCl, 0.5% (vol/vol) Nonidet P-40 (NP-40), 1 mM NaF, 1 mM dithiothreitol (DTT), 1 mM phenylmethylsulfonyl fluoride (PMSF), EDTA-free protease inhibitors (Thermo Fisher Scientific (Heysham), Lancashire, UK), and RNase inhibitor (1 unit/mL). Following a 15 min incubation on ice, the cell lysate was centrifuged at 10,000 rpm for 20 min at 4 °C. For each anti-Myc immunoprecipitation, 50 μL of packed Pierce anti-c-Myc agarose beads (Thermo Fisher Scientific (Heysham), Lancashire, UK) were prewashed and incubated with 1 mL of whole-cell lysate (1 to 2 mg/mL) for 2 h with rotation at 4 °C. Following six washes (each for 5 min) using a low-stringency buffer (containing 1 mM PMSF, 0.1% [vol/vol] Tween 20, and 0.1% [vol/vol] NP-40) at 4 °C, the IP-bound proteins were released by adding 50 μL of protein-loading buffer and heating at 95 °C for 5 min.

### 2.7. In-Gel Proteolysis and MS Analysis

Gel-based proteolysis was performed according to a previously described method [24]. Initially, the proteins in the gel were reduced with 3 mM DTT at 60 °C for 30 min, followed by alkylation with 10 mM iodoacetamide in 100 mM ammonium bicarbonate in a light-protected environment at room temperature for 30 min. The proteins were then digested enzymatically with modified trypsin (Promega, Beit Haemek, Israel) in a solution containing 10% acetonitrile and 10 mM ammonium bicarbonate at an enzyme-to-substrate ratio of 1:10, overnight at 37 °C. A secondary digestion with trypsin was performed for an additional 4 h at 37 °C. The resultant tryptic peptides were desalted using homemade C18 stage tips, dried, and re-suspended in 0.1% formic acid. These peptides were further separated by reverse-phase chromatography on a 0.075 × 300 mm fused silica capillary column (J&W, Agilent Technologies, Santa Clara, CA, USA) packed with Reprosil reversed-phase material (Dr Maisch GmbH, Ammerbuch, Germany). Peptide elution was achieved using a linear gradient of 5% to 28% acetonitrile with 0.1% formic acid in water over 60 min, followed by a 15 min gradient of 28% to 95% acetonitrile with 0.1% formic acid in water, and finally, 15 min at 95% acetonitrile with 0.1% formic acid in water, at a flow rate of 0.15 μL/min. Mass spectrometry analysis was conducted using a QExactive Plus mass spectrometer (Thermo Fisher Scientific, Waltham, MA, USA) operating in positive mode. The procedure involved repetitive full MS scans followed by high collision dissociation (HCD) of the 10 most abundant ions selected from the initial MS scan.

For the data analysis, MaxQuant software version 2.1.1.0 [25] was used, facilitating peak picking and identification through the Andromeda search engine. The search was conducted against the *E. histolytica* section of the UniProt database, with a mass tolerance of 4.5 ppm for precursor ions and 20 ppm for fragment ions. The accepted variable modifications included the oxidation of methionine, protein N-terminus acetylation, and biotin on lysine, while the carbamidomethylation of cysteine was considered static. Peptides with a minimum length of seven amino acids and up to two miscleavages were allowed. Label-free quantification was performed using the same software, with the peptide- and protein-level false discovery rates (FDRs) filtered to 1% using the target–decoy strategy. The protein tables were filtered to remove identifications from the reverse database and common contaminants. Statistical analysis of the identification and quantification results was performed using Perseus software (Perseus 1.6.7) from Mathias Mann’s group. Proteins were considered significantly altered if they exhibited at least a 2-fold change in abundance in the Myc-tagged EhDUF2419 strain compared to the control, with a *p*-value < 0.05 and razor + unique peptides > 1.

### 2.8. PANTHER Classification System

The PANTHER Classification System (Version 18.0) was employed for the data analysis in this study, accessed via http://pantherdb.org/ (accessed on 20 October 2023) [26]. To categorize the proteins, the “protein class” ontology setting was utilized. The statistical over-representation test was carried out using default settings, employing the annotation dataset corresponding to the PANTHER protein class and opting for FDR correction for multiple testing.

### 2.9. Ribosome Purification

Ribosomes were pelleted according to the method described in [27,28], with some modifications. For the experiments involving Q treatment, the trophozoites were incubated with 0.1 μM Q for 2 days prior to collection. Briefly, 1 × 10^7^ cells were treated with 0.1 mg/mL cycloheximide (CHX) for 5 min and then harvested. Following collection, the cells were lysed on ice for 10 min using lysis buffer containing 20 mM Hepes (pH 7), 100 mM KCl, 5 mM MgCl_2_, 0.1% Triton, 2 mM DTT, proteinase inhibitor, and RNase inhibitor. DNase treatment was carried out at 25 °C for 5 min. Nuclei were removed by centrifugation at maximum speed for 10 min at 4 °C, and the resulting supernatant was collected. Ribosomes were then isolated by pelleting with a sucrose cushion (prepared with 10 mL lysis buffer and 12.5 mL of a solution containing 20 mM HEPES pH 7, 100 mM KCl, 5 mM MgCl_2_, and 0.5 M sucrose) through ultracentrifugation at 60,000 RPM for 1 h and 50 min. The obtained ribosome pellets were subsequently resuspended in 20 μL of lysis buffer and boiled at 95 °C for 5 min. The samples were loaded onto a 12% SDS-PAGE gel and subjected to immunoblotting using a mouse anti-Myc antibody (Cell Signaling Technology, Danvers, MA, USA) at a 1:1000 dilution and a homemade Ribosomal 60S subunit protein L1A (RPL1A) antibody at a 1:1000 dilution (kindly supplied by Prof. Mordechai Choder from Technion, Haifa, Israel) in *Saccharomyces cerevisiae*. RPL1A in *Saccharomyces cerevisiae* exhibits a 53% sequence identity with 60S ribosomal protein L10a-2 (EHI_012480) in *E. histolytica* HM-1:IMSS. In this study, EHI_012480 was significantly enriched in the pulldown proteins that interact with EhDUF2419.

### 2.10. Assessment of Protein Synthesis Through Surface Sensing of Translation (SUnSET)

Protein synthesis in the trophozoites was evaluated using the SUnSET method [29,30]. For the experiments involving Q treatment, the trophozoites were incubated with 0.1 μM Q for 2 days prior to collection. Briefly, the trophozoites (2 × 10^6^) were incubated with 10 μg/mL puromycin (Merck, Rehovot, Israel), a structural analog of tyrosyl-tRNA, for 20 min at 37 °C. After incubation, the trophozoites were lysed using 1% Igepal (Merck, Rehovot, Israel) in PBS. The proteins were separated on a 10% SDS-PAGE gel in SDS-PAGE running buffer and transferred to a nitrocellulose membrane in protein transfer buffer. To ensure equal loading, the membrane was stained with Ponceau-S (Merck, Rehovot, Israel) before immunostaining. Puromycin incorporation was detected by immunoblotting with a 1:1000 dilution of monoclonal puromycin antibody (12D10 clone, Merck Millipore, Rosh-Ha’ayin, Israel). After the primary antibody incubation, the blots were treated with a 1:5000 dilution of secondary antibody (Jackson ImmunoResearch, West Grove, PA, USA) for 2 h at room temperature. Enhanced chemiluminescence (WesternBright™ ECL, Advansta, CA, USA) was used for development, and the blots were photographed using a Fusion FX7 Edge Spectra, Fusion-Vilber, Collegien, France. Protein synthesis quantification was performed by measuring the intensity of the immunoreactive blots (densitometry) with Fiji software version 1.54f [31].

### 2.11. N-Acryloyl-3-Aminophenylboronic Acid (APB) Northern Blotting for E. histolytica tRNA^His^_GUG_

Gels containing acryloyl aminophenylboronic acid were prepared, with slight modifications, based on the method reported by Igloi and Kossel [32]. For the experiments involving Q treatment, the trophozoites were incubated with 0.1 μM Q for 2 days prior to extraction. In brief, 15 μg of RNA was deacetylated in 100 mM Tris-HCl (pH 9) for 30 min at 37 °C. The RNA was then precipitated using ethanol and resuspended in 10 mL DEPC-treated water. The samples were denatured for 10 min at 70 °C and subsequently run on Tris-acetate EDTA (TAE) buffer gels at 4 °C. These gels contained 8 M urea, 15% acrylamide, and 5 mg/mL aminophenylboronic acid (Merck, Rehovot, Israel) and were run using a Bio-Rad mini gel system at 75 V for 7 h until the bromophenol blue dye reached the gel’s bottom. Post-electrophoresis, the gels were stained with ethidium bromide in 1× TAE buffer for 20 min to verify the equal sample loading, then destained with ultrapure water for another 20 min. The samples were transferred onto a Hydrobond-XL membrane (GE Healthcare, Chicago, IL, USA) via electrotransfer in 0.5 × TAE buffer for 45 min at 150 V. The membrane was UV cross-linked using a Stratalinker UV crosslinker 1800, Stratagene, La Jolla, CA, USA set to 120 mJ, followed by two 15 min hybridizations in 5 mL of hybridization buffer (20 mM sodium phosphate buffer [pH 7.3], 300 mM NaCl, 1% SDS). Next, 150 μg/mL heat-denatured salmon sperm DNA (ssDNA) was added to the buffer for blocking, performed at 60 °C for 1 h. The membrane was incubated with 15 pmol biotinylated tRNA probes targeting tRNA^His^_GUG_ at 60 °C for 16 h, then washed for 10 min in wash buffer (20 mM sodium phosphate buffer [pH 7.3], 300 mM NaCl, 2 mM EDTA, 0.5% SDS) at 60 °C. An additional 10 min incubation in hybridization buffer at room temperature was followed by a 30 min incubation with streptavidin–horseradish peroxidase (HRP) conjugate in 5 mL hybridization buffer (diluted 1:5000). Subsequent washes were performed twice for 10 min each in wash buffer. The membrane was then treated with enhanced chemiluminescence reagent (WesternBright^TM^ ECL, Advansta, CA, USA) and photographed with Fusion FX7 Edge Spectra.

### 2.12. Immunofluorescence Microscopy

Trophozoites (1.5 × 10^5^/mL) were resuspended in serum-free TYI medium at 37 °C and placed onto glass coverslips pre-cleaned with acetone, positioned at the bottom of the wells in a 24-well plate. The trophozoites were allowed to attach to the coverslips by incubating them for 1 h at 37 °C. After adhesion, they were fixed using 4% paraformaldehyde (PFA, prewarmed to 37 °C, Electron Microscopy Sciences, Hatfield, PA, USA) for 30 min at room temperature. Subsequently, the cells were permeabilized for 1 min with 0.1% Triton X-100/PBS at room temperature. The coverslips were washed three times with PBS and quenched with PBS containing 50 mM NH_4_Cl for 30 min at room temperature. Following quenching, the coverslips were blocked using 1% bovine serum albumin (BSA, MP Biomedicals, Ohio, USA) in PBS (BSA/PBS) for 1 h at room temperature. The samples were incubated overnight with a 1:1000 dilution of anti-Myc antibody (Cell Signaling Technology, Danvers, MA, USA) and an anti-RPL1A (kindly supplied by Prof. Mordechai (Motti) Choder from Technion, Haifa, Israel) antibody. The following day, the samples were washed in PBS and 1% BSA/PBS washes and incubated for 4 h at 4 °C with Alexa Fluor 488 (1:250 dilution, Jackson ImmunoResearch, PA, USA), Rhodamine Red™-X (1:250 dilution, Jackson ImmunoResearch, PA, USA), and 4′,6-diamidino-2-phenylindole (DAPI; 1:1000 dilution, MP Biomedicals, OH, USA). After incubation, the coverslips were washed again in PBS. After further washing steps, they were mounted on microscope slides using Fluoromount G, SouthernBiotech, Alabama, USA. Finally, the samples were visualized with a confocal immunofluorescence microscope (ZEISS-LSM700 Meta Laser Scanning System, Zeiss LSM700, Oberrochen, Germany) equipped with a 63× oil immersion objective, and the fluorescence intensity was quantified using Fiji software version 1.54f [31].

For the quantification of the co-localization, whole regions of singly labeled cells were selected to set the thresholds. Then, the regions were used for pixel quantification. The co-localization of MycEhDUF2419 and RPL1A was quantified using Zeiss Zen software (Black Edition), version 3.5, which calculates the overlap and co-localization coefficient as derived from Pearson’s correlation coefficient (PCC) and Manders’ overlap coefficient (MOC) [33,34].

### 2.13. Statistical Analysis

The statistical analysis and graphical representations were performed using Prism 9 (GraphPad Software Inc., San Diego, CA, USA). The data are expressed as the mean ± standard error of the mean (SEM) from 2 to 4 biological replicates. Unless stated otherwise, significance was determined using one-way ANOVA for multiple comparisons and the unpaired t-test for comparisons between two groups.

## 3. Results

### 3.1. Interactome of MycEhDUF2419 in E. histolytica Trophozoites

**Figure 1:**
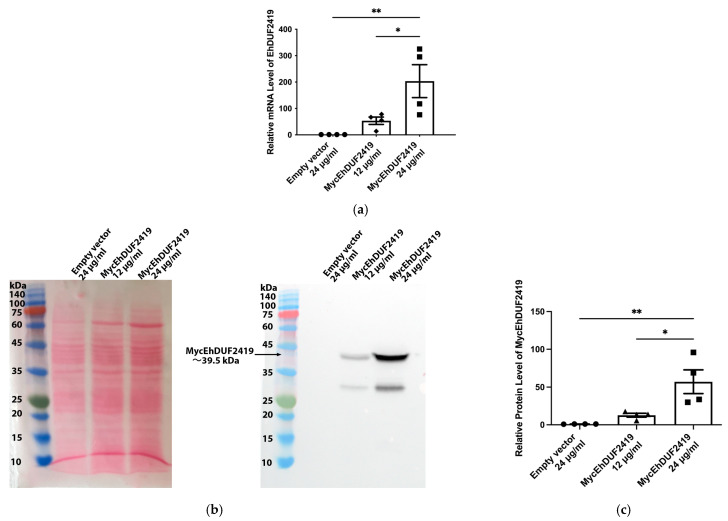
Overexpression of MycEhDUF2419 in *E. histolytica* trophozoites. (**a**) Quantification of the EhDUF2419 mRNA expression levels in empty vector (24 μg/mL) and MycEhDUF2419 overexpression trophozoites (MycEhDUF2419 12 μg/mL, MycEhDUF2419 24 μg/mL) was performed by qPCR. The relative fold change was calculated using the 2^−∆∆Ct^ method. The data represent the mean ± SEM of three independent experiments, each with one to two technical replicates. Statistical significance is denoted by asterisks (* *p* < 0.05, ** *p* < 0.01). (**b**) Left: Ponceau S stain showing the total protein labeling. Right: Western blotting was performed on the total protein extracts prepared from empty vector (24 μg/mL) and MycEhDUF2419 overexpression trophozoites (MycEhDUF2419 12 μg/mL, MycEhDUF2419 24 μg/mL). The proteins were separated on 12% SDS-PAGE gels and analyzed by Western blotting using a Myc antibody (1:1000). The trophozoites were grown with 12 μg/mL or with 24 μg/mL G418. (**c**) The MycEhDUF2419 signal was normalized relative to the total protein signal using Fiji software version 1.54f. The data represent the mean ± SEM of three independent experiments, each with one to two technical replicates. Statistical significance is denoted by asterisks (* *p* < 0.05, ** *p* < 0.01).

We have previously identified that EhDUF2419 acts as a queuine salvage enzyme in *E. histolytica* by catalyzing the conversion of Q into queuine [16]. However, the interaction networks, or interactome, of EhDUF2419 within the parasite remain largely unexplored. To investigate the interactome of EhDUF2419, we transfected *E. histolytica* trophozoites with a plasmid encoding Myc-tagged EhDUF2419. We induced the overexpression of EhDUF2419 by increasing the plasmid copy number, which was achieved by elevating the concentration of the antibiotic G418. The plasmid copy number is directly proportional to the level of resistance of the cultures to G418, allowing us to control the level of EhDUF2419 expression [35]. We quantified the EhDUF2419 mRNA expression levels in trophozoites carrying the pKT-3M vector (empty vector) and Myc-tagged EhDUF2419 overexpressing trophozoites using qPCR. Our results showed a significant increase in the EhDUF2419 mRNA levels in trophozoites treated with 24 μg/mL G418 compared to both empty vector trophozoites and Myc-tagged EhDUF2419 overexpressing trophozoites cultivated with 12 μg/mL G418 (Figure 1a). Western blot analysis revealed a distinct 39.5 kDa band corresponding to MycEhDUF2419 in Myc-tagged EhDUF2419-overexpressing trophozoites cultivated with 24 μg/mL G418 (Figure 1b). In contrast, MycEhDUF2419 was expressed with less intensity in trophozoites cultivated with 12 μg/mL G418 (Figure 1b,c), which aligns with the lower levels of Myc-tagged EhDUF2419 mRNA observed at this concentration (Figure 1a). Additionally, the Myc antibody detected a 30 kDa band of unknown origin, which may result from non-specific binding or degradation of the Myc-tagged EhDUF2419 recombinant protein (Figure 1b). Overall, Western blot analysis using a Myc antibody confirmed the overexpression of the EhDUF2419 protein in Myc-tagged EhDUF2419-overexpressing trophozoites.

Based on the optimal expression level of Myc-tagged EhDUF2419, we selected transfectants treated with 24 μg/mL G418 for the pulldown of EhDUF2419. This process involved using anti-c-Myc agarose beads for immunoprecipitation, followed by mass spectrometry-based proteomics analysis.

**Figure 2:**
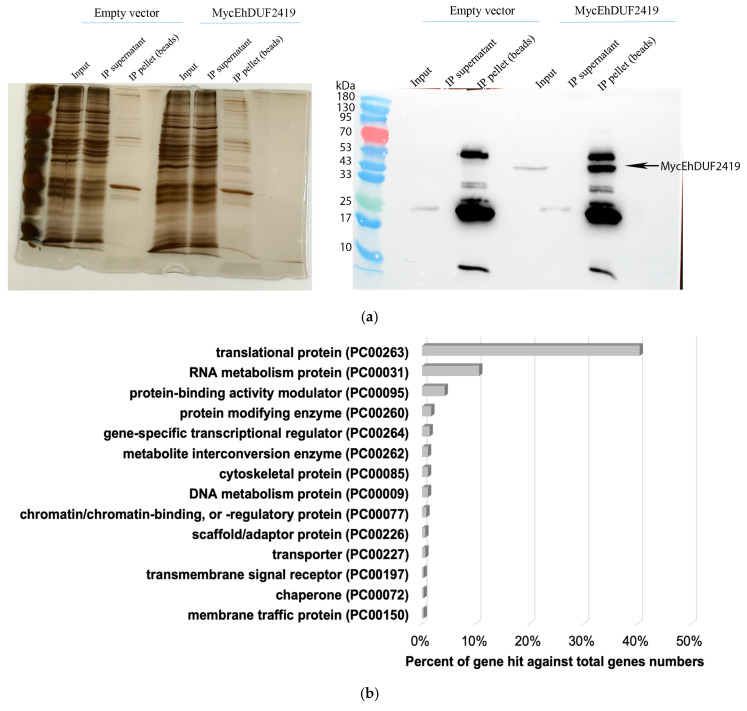

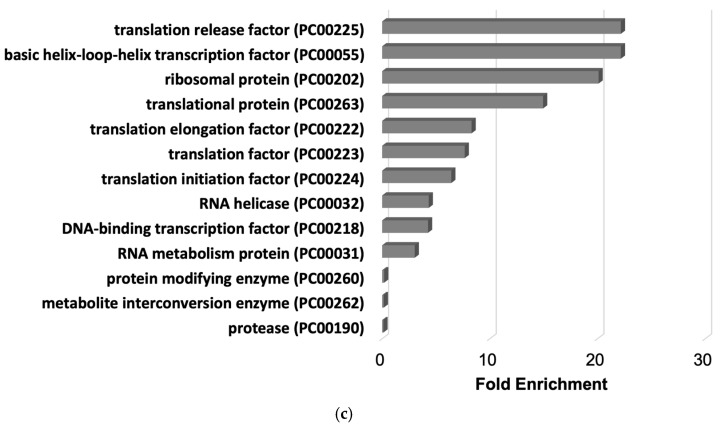
Pulldown of EhDUF2419 by anti-Myc IP and proteomics analysis. (**a**) Silver staining (left) and Western blotting (right) were used to assess the anti-Myc IP samples prior to mass spectrometry analyses. The anti-Myc Western blot shows a Myc signal in the IP elution lanes for MycEhDUF2419 24 μg/mL trophozoites but not in the empty vector trophozoites, indicating specific pulldown of the Myc-tagged protein. The arrow indicates the detected protein band for EhDUF2419 in the IP elution samples. (**b**) Triplicate IP samples were subjected to in-gel trypsin digestion for LC-MS/MS-based proteomics analysis. The PANTHER classification (http://pantherdb.org (accessed on 20 October 2023) of the 335 proteins identified in the pulldown of MycEhDUF2419-overexpressing trophozoites compared to the empty vector trophozoites is shown. (**c**) PANTHER fold enrichment analysis was performed on the 335 proteins identified in MycEhDUF2419-overexpressing trophozoites compared to empty vector trophozoites.

The protein enrichment post-immunoprecipitation was verified using Western blotting and silver staining. The successful pulldown of the Myc-tagged EhDUF2419 protein in the IP sample confirmed that the tagged protein was successfully immunoprecipitated (Figure 2a). In contrast, no Myc-tagged EhDUF2419 protein was detected in the IP product of empty vector trophozoites (Figure 2a). Lysates prepared from three biological replicates of empty vector trophozoites and Myc-tagged EhDUF2419-overexpressing trophozoites were immunoprecipitated. Pulled-down proteins were eluted from the beads with SDS-PAGE sample-loading buffer, separated by SDS-PAGE, and subjected to LC-MS/MS analysis following in-gel trypsin digestion. We identified 335 proteins that potentially interact with EhDUF2419 (*p*-value < 0.05, fold change > 2) in the Myc-tagged EhDUF2419-overexpressing trophozoites (Table S1). These 335 proteins were categorized using the PANTHER classification system [26] (Figure 2b). The six most prevalent protein families included translational protein (PC00263) (exemplified by 60S ribosomal protein L10 [EHI_044810]), RNA metabolism protein (PC00031) (exemplified by tRNA (Cytosine-5-)-methyltransferase, EhDNMT2 [EHI_103830]), protein-binding activity modulator (PC00095) (exemplified by Rho guanine nucleotide exchange factor EHI_159500), protein-modifying enzyme (PC00260) (exemplified by E3 ubiquitin-protein ligase listerin [EHI_190430]), gene-specific transcriptional regulator (PC00264) (exemplified by eukaryotic translation initiation factor 4 gamma [EHI_044930]) and metabolite interconversion enzyme (PC00262) (exemplified by glycylpeptide N-tetradecanoyltransferase [EHI_159730). The PANTHER statistical overrepresentation test, which compares classifications of multiple clusters to a reference list, revealed significant enrichment (*p*-value and q-value < 0.05) for proteins annotated as translation release factor (PC00225) (exemplified by eukaryotic peptide chain release factor subunit 1 [EHI_152750]), basic helix-loop-helix transcription factor (PC00055) (exemplified by eukaryotic translation initiation factor 4 gamma [EHI_044930]), ribosomal protein (PC00202) (exemplified by 60S ribosomal protein L10 [EHI_044810]), translational protein (PC00263) (exemplified by eukaryotic translation initiation factor 3 30 kDa subunit [EHI_178890]), translation elongation factor (PC00222) (exemplified by elongation factor 2 [EHI_155660]), translation factor (PC00223) (exemplified by WD domain containing protein [EHI_118720]), translation initiation factor (PC00224) (exemplified by eukaryotic translation initiation factor eIF-5 [EHI_129770]), RNA helicase (PC00032) (exemplified by helicase [EHI_053600]) and so on (Figure 2c).

Moreover, we analyzed the functional enrichment of these 335 proteins, which were previously identified as potentially interacting with EhDUF2419 using LC-MS/MS, with STRING, a database of known and predicted protein–protein interactions (https://string-db.org/ (accessed on 4 September 2024)) (https://version-12-0.string-db.org/cgi/network?networkId=bxOIiARHdZwO (accessed on 4 October 2024), and Table S2). Subsequently, MCL clustering (inflation parameter = 3) was performed, resulting in 12 clusters. Notably, the largest cluster, comprising 236 proteins, was characterized as ribosome-associated proteins.

Meanwhile, STRING predicts possible interactions between EhDUF2419 and EhTGT (EHI_035660), EhDUF2419 and a pseudouridine synthase and archaeosine transglycosylase domain RNA-binding motif containing protein (EHI_016460), EhDUF2419 and an endonuclease (EHI_134360) and so on (Table S3). Notably, the pseudouridine synthase and archaeosine transglycosylase domain RNA-binding motif-containing protein (EHI_016460) is one of the proteins identified in the EhDUF2419 interactome (Table S1).

### 3.2. EhDUF2419 Co-Purified with Ribosomal Proteins in the Ribosome Fraction

**Figure 3:**
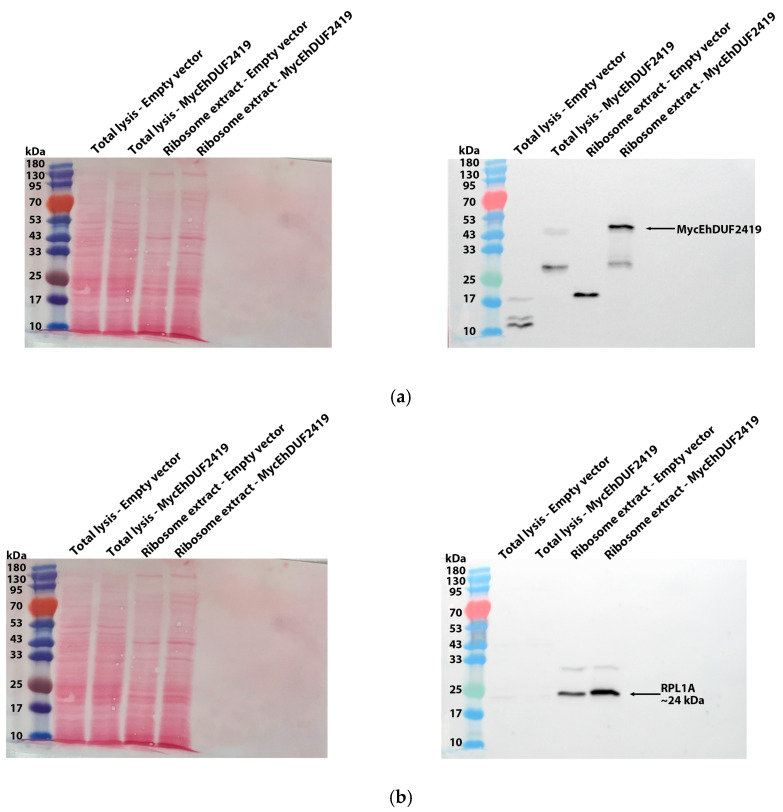
Co-purification of MycEhDUF2419 with ribosomal proteins. (**a**,**b**) Left: Ponceau S stain showing the total protein labeling. Right: Total lysates prepared from control *E. histolytica* trophozoites (empty vector, 24 μg/mL) and MycEhDUF2419 overexpression trophozoites (24 μg/mL) were subjected to ultracentrifugation through a sucrose cushion to pellet the ribosomes. The resulting pellets were resuspended, boiled, and loaded onto a 12% SDS-PAGE gel for immunoblotting with a Myc antibody (**a**) or RPL1A antibody (**b**). The experiment was repeated independently three times, with similar results.

The identification of ribosomal and other translation-related proteins in the anti-c-Myc agarose bead immunoprecipitation (Figure 2b,c) indicates that EhDUF2419 interacts with ribosomal proteins and may play a role in regulating translation in the parasite. To validate this hypothesis, we purified ribosomes and checked if EhDUF2419 co-purified with them. Our results showed that EhDUF2419 was significantly enriched in the ribosome fraction (Figure 3a). Additionally, we verified that one of the ribosomal proteins, RPL1A (corresponding to the 60S ribosomal protein L10a-2 in *E. histolytica*, EHI_012480), identified among the proteins significantly enriched following the pulldown of MycEhDUF2419, was also present in the ribosome fraction (Figure 3b). These results strongly suggest that EhDUF2419 interacts with ribosomal proteins.

**Figure 4:**
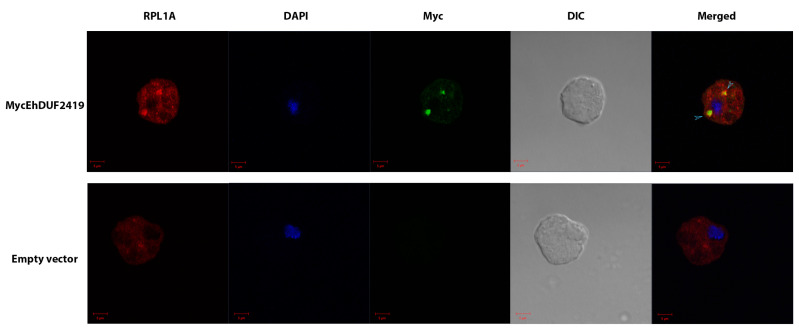
Co-localization of MycEhDUF2419 and ribosomal protein RPL1A in transfected trophozoites visualized by immunofluorescence microscopy. Confocal laser scanning microscopy employing a 63× oil immersion lens objective was used to examine the subcellular localization of MycEhDUF2419 and ribosomal protein RPL1A in *E. histolytica* trophozoites transfected with MycEhDUF2419 or an empty vector. MycEhDUF2419 (green) was detected using a primary anti-Myc antibody followed by an Alexa Fluor 488-conjugated secondary antibody, while RPL1A (red) was visualized using a primary anti-RPL1A antibody followed by a Rhodamine Red™-X secondary antibody. Co-localization of MycEhDUF2419 and RPL1A is indicated by the light blue arrows in the merged image. Nuclei were stained blue using DAPI dye. The MOC and PCC were used to analyze the co-localization of MycEhDUF2419 and RPL1A in 20 MycEhDUF2419-transfected *E. histolytica* trophozoites, using Zeiss Zen software (Black Edition), version 3.5. Data are presented as the mean ± SEM, with MOC values of 0.9093 ± 0.01547 and PCC values of 0.4075 ± 0.05458.

To determine the subcellular localization of MycEhDUF2419 and confirm its interaction with the ribosomal protein RPL1A, we performed immunofluorescence microscopy to co-stain MycEhDUF2419 and RPL1A. In trophozoites transfected with MycEhDUF2419, the results revealed that MycEhDUF2419 was predominantly localized in the cytoplasm, where it co-localized with RPL1A, a known cytoplasmic protein [36]. In contrast, no MycEhDUF2419 signal was detected in trophozoites transfected with the empty vector, although RPL1A was still present (Figure 4). The high Manders’ overlap coefficient (MOC) close to one suggests that the proteins are highly co-localized in terms of the spatial location. However, the Pearson’s correlation coefficient (PCC) of 0.4 suggests that their intensities are moderately correlated, implying different expression or concentration levels—where one protein could be more abundant in specific regions, affecting the PCC without diminishing the spatial overlap (Figure 4).

### 3.3. EhDUF2419 Interacts with Ribosomal Protein Independent of Its Catalytic Domain

**Figure 5:**
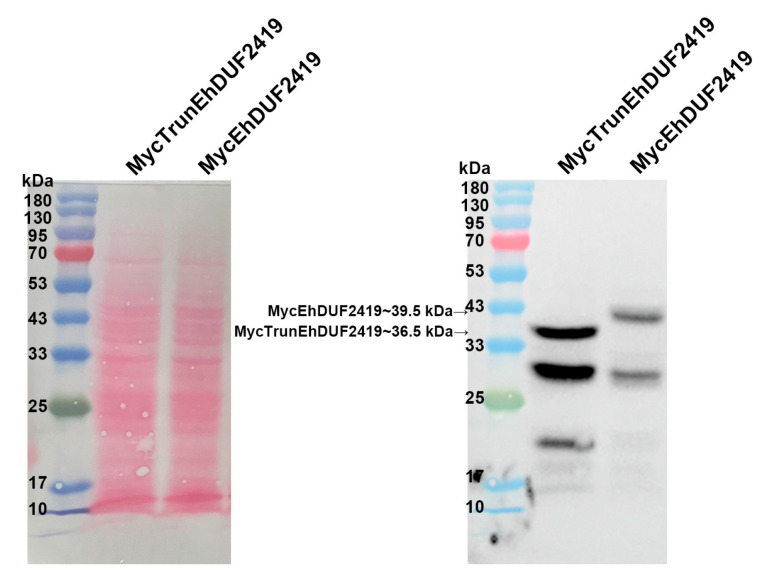
Overexpression of truncated EhDUF2419 in *E. histolytica* trophozoites. Left: Ponceau S stain showing the total protein labeling. Right: Western blotting was performed on the total protein extracts prepared from truncated EhDUF2419 overexpression trophozoites (MycTrunEhDUF2419, 24 μg/mL) and MycEhDUF2419 overexpression trophozoites (24 μg/mL). The proteins were separated on 12% SDS-PAGE gels and analyzed by Western blotting using a Myc antibody (1:1000).

Given that some proteins, such as Shiga-like toxin 1 [37] or protein-arginine methyltransferase 5 (PRMT5) [38], interact with ribosomal proteins through their catalytic sites, we decided to construct a catalytically inactive MycEhDUF2419 plasmid to investigate whether EhDUF2419 interacts with ribosomal proteins in a manner dependent on its catalytic domain. The active site of the DUF2419 protein was deduced based on the StDUF2419 protein from *Sphaerobacter thermophilus* [18]. Sequence alignment showed that EhDUF2419 shares 33% identity with StDUF2419, with the active site residues D298 and W302 in StDUF2419 corresponding to D280 and W284 in EhDUF2419 (Figure S1a). We used AlphaFold [39,40] to predict the structure of EhDUF2419 and mapped the active site and ligand in the crystal structure of StDUF2419 (PBD: 7U91, [17]) (Figure S1b–d). We truncated EhDUF2419 between residue D280 and the stop codon, resulting in the deletion of 26 amino acids, including part of the active pocket. We then constructed a plasmid expressing this truncated version of EhDUF2419 with a Myc-tag and transfected it into *E. histolytica* trophozoites. The MycTrunEhDUF2419 transfectants were cultivated in the presence of 24 μg/mL G418. Western blot analysis using a Myc antibody revealed a distinct 36.5 kDa band corresponding to the expected size of the MycTrunEhDUF2419 in MycTrunEhDUF2419-overexpressing trophozoites, different from the 39.5 kDa full-length MycEhDUF2419 (Figure 5).

**Figure 6:**
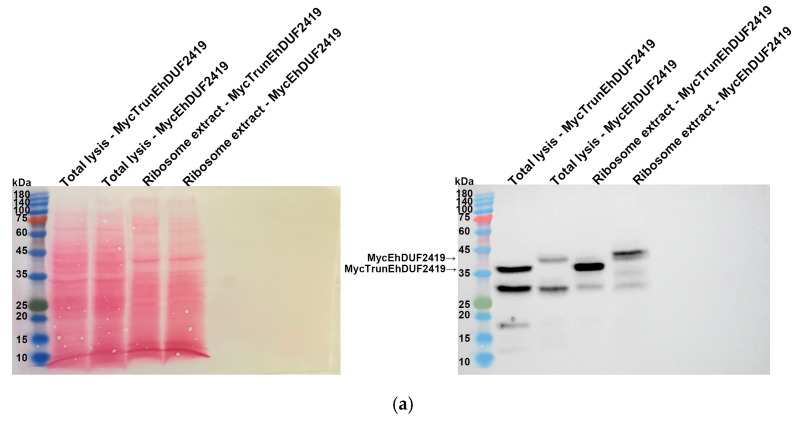

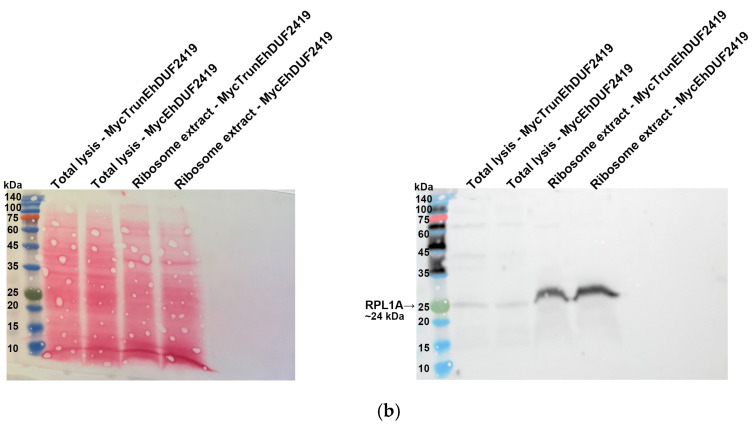
Co-purification of MycTrunEhDUF2419 with ribosomal proteins. (**a**,**b**) Left: Ponceau S stain showing the total protein labeling. Right: The total lysates prepared from MycTrunEhDUF2419 overexpression trophozoites (24 μg/mL) and MycEhDUF2419 overexpression trophozoites (24 μg/mL) were subjected to ultracentrifugation through a sucrose cushion to pellet the ribosomes. The resulting pellets were resuspended, boiled, and loaded onto a 12% SDS-PAGE gel for immunoblotting with a Myc antibody (**a**) or RPL1A antibody (**b**). The experiment was repeated independently three times, with similar results.

To investigate whether the interaction between EhDUF2419 and ribosomal proteins depends on its active site, we purified the ribosomes and examined whether the MycTrunEhDUF2419 co-purified with them. The co-detection of the MycTrunEhDUF2419 (Figure 6a) and the ribosomal protein RPL1A (Figure 6b) in the ribosome-enriched fraction strongly suggests that the EhDUF2419 catalytic domain is not necessary for its interaction with ribosomal proteins.

### 3.4. EhDUF2419 Overexpression Reduces Q-tRNA Formation in Presence of Q

**Figure 7:**
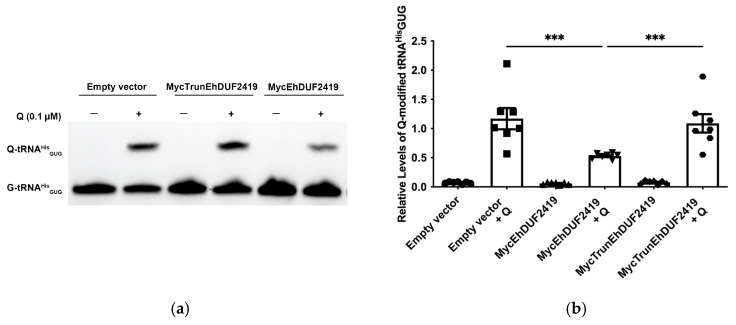
Effect of full-length and truncated EhDUF2419 overexpression on the Q-modified tRNA^His^_GUG_ levels in *E. histolytica* trophozoites. (**a**) APB northern blot analysis of tRNA^His^_GUG_ in control, MycEhDUF2419 and MycTrunEhDUF2419 overexpression trophozoites following cultivation with Q. Trophozoites were cultivated in the presence of 0.1 μM Q for 2 days. (**b**) Quantitative relative levels of Q-modified tRNA^His^_GUG_. The data represent the mean ± SEM of four independent experiments, each with one to two technical replicates. Statistical significance is denoted by asterisks (*** *p* < 0.001).

EhDUF2419 plays a crucial role in salvaging queuine from Q, which is subsequently incorporated into tRNA by EhTGT to form Q-tRNA [9,16]. Since Q is limited in the *E. histolytica* culture medium (Figure 7 and [16]), we hypothesized that overexpression of EhDUF2419 without additional Q in the culture media of the parasite would not affect the Q-tRNA levels. However, in the presence of supplemental Q, we expected that overexpression of EhDUF2419 would increase the queuine availability, leading to higher Q-tRNA formation. To assess this, we examined the effect of EhDUF2419 overexpression on the Q-tRNA^His^_GUG_ levels using APB polyacrylamide gel analysis in MycTrunEhDUF2419, in MycEhDUF2419 transfectants, as well as in controls with or without additional Q. The APB gel method detects Q-tRNA using northern blots by exploiting the cis-diol group on the Q modification, which slows the migration of Q-modified tRNA compared to unmodified tRNA in the presence of APB [41]. The results showed that prior to the addition of Q to the culture medium, the Q-tRNA^His^_GUG_ levels in the *E. histolytica* trophozoites were nearly undetectable across all the experimental conditions. However, upon supplementation with Q, the Q-tRNA^His^_GUG_ levels in trophozoites overexpressing MycEhDUF2419 were significantly lower compared to the controls and MycTrunEhDUF2419-overexpressing trophozoites. However, no significant difference was observed between the controls and the trophozoites overexpressing MycTrunEhDUF2419 (Figure 7).

**Figure 8:**
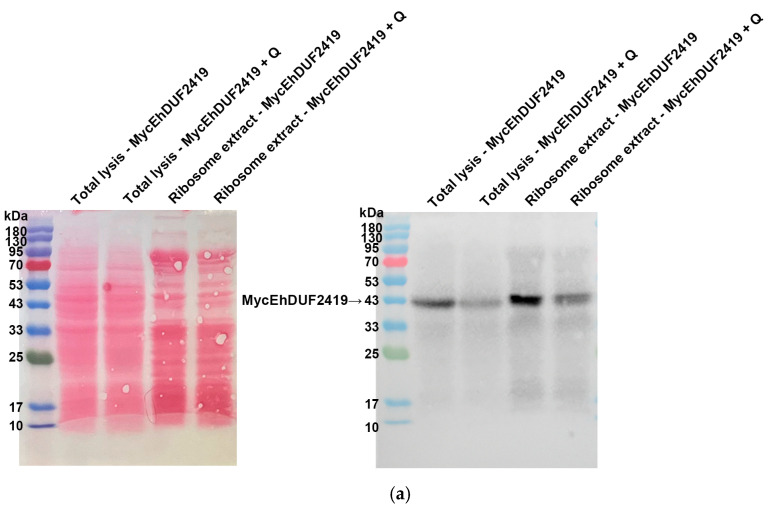

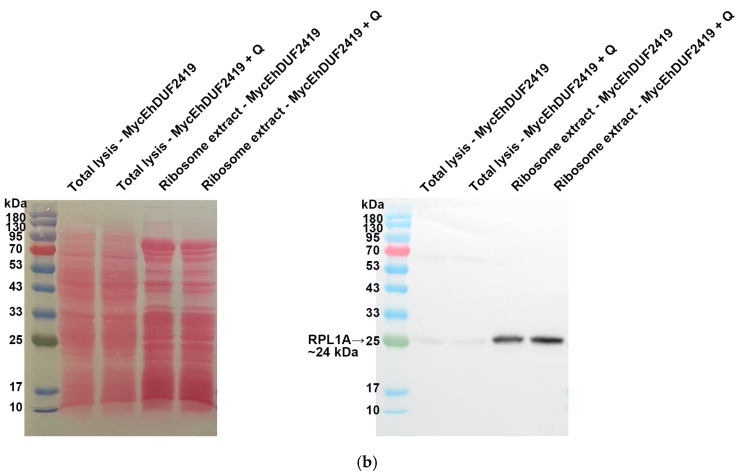
Co-purification of MycEhDUF2419 with ribosomal proteins, with or without Q treatment. (**a**,**b**) Left: Ponceau S stain showing the total protein labeling. Right: The total lysates were prepared from MycEhDUF2419 overexpression trophozoites (24 μg/mL) treated with or without Q. The lysates were subjected to ultracentrifugation through a sucrose cushion to pellet the ribosomes. The resulting pellets were resuspended, boiled, and loaded onto a 12% SDS-PAGE gel for immunoblotting with either a Myc antibody (**a**) or an RPL1A antibody (**b**). The experiment was repeated independently twice, with similar results.

**Figure 9:**
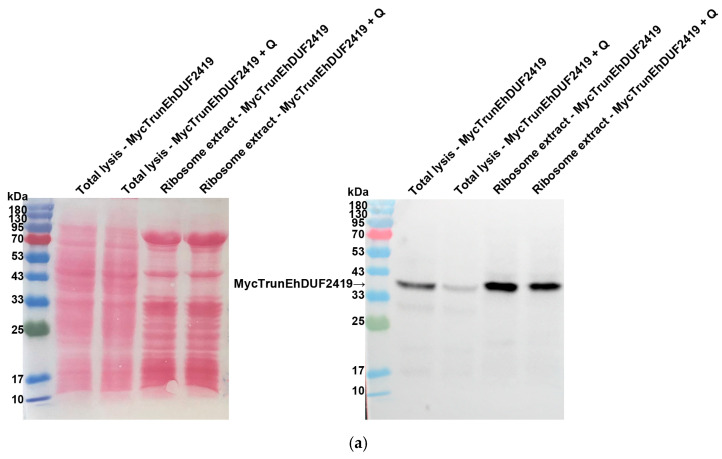

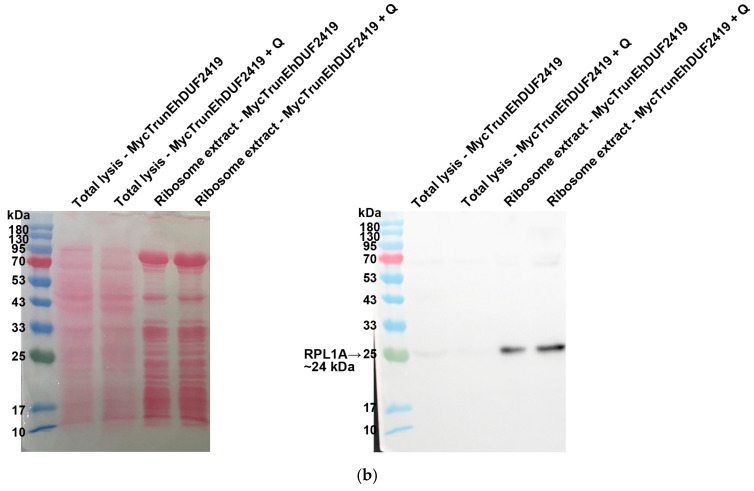
Co-purification of MycTrunEhDUF2419 with ribosomal proteins, with or without Q treatment. (**a**,**b**) Left: Ponceau S stain showing the total protein labeling. Right: The total lysates were prepared from MycTrunEhDUF2419 overexpression trophozoites (24 μg/mL) treated with or without Q. The lysates were subjected to ultracentrifugation through a sucrose cushion to pellet the ribosomes. The resulting pellets were resuspended, boiled, and loaded onto a 12% SDS-PAGE gel for immunoblotting with either a Myc antibody (**a**) or an RPL1A antibody (**b**). The experiment was repeated independently twice, with similar results.

Since the addition of Q altered the Q-tRNA levels in MycEhDUF2419 transfectants (Figure 7), we wanted to determine whether the interaction between EhDUF2419 and ribosomal proteins is influenced by Q treatment. We purified the ribosomes and assessed the co-purification of MycEhDUF2419 and MycTrunEhDUF2419 with ribosomal proteins under both Q-treated and untreated conditions. The presence of MycEhDUF2419 and MycTrunEhDUF2419 in the ribosome-enriched fractions of trophozoites, regardless of Q treatment (Figure 8a and Figure 9a), along with the co-detection of ribosomal protein RPL1A (Figure 8b and Figure 9b), strongly suggests that the interaction between EhDUF2419 and ribosomal proteins is independent of Q treatment.

EhDUF2419 is involved in the production of Q-tRNA, which subsequently regulates the translational speed and fidelity in eukaryotes [10,11] and influences the translation of stress-related proteins in *E. histolytica* [9]. This suggests that EhDUF2419 indirectly influences translation through Q-tRNA modification. However, our results strongly suggest that EhDUF2419 interacts with ribosomal proteins, indicating a more direct role for EhDUF2419 in regulating translation. To investigate this, we employed the SUnSET method [42] to monitor the changes in protein synthesis in control trophozoites, MycEhDUF2419 and MycTrunEhDUF2419 overexpression trophozoites, both with or without Q treatment. Our results revealed no significant differences in protein synthesis between the control, MycEhDUF2419, and MycTrunEhDUF2419-overexpressing strains, regardless of Q treatment (Figure S2).

### 3.5. EhDUF2419 Is Regulated by Proteasome-Mediated Degradation Pathways

**Figure 10:**
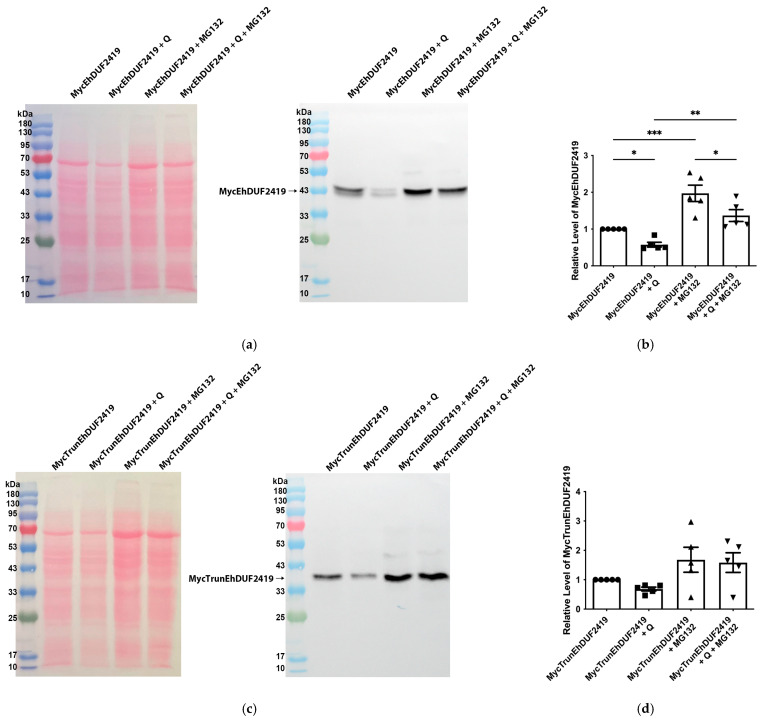
Regulation of MycEhDUF2419 levels by proteasome-mediated degradation. (**a**) Left: Ponceau S stain showing the total protein labeling. Right: Western blotting was performed on the total protein extracts prepared from MycEhDUF2419 overexpression trophozoites (24 μg/mL) treated with or without Q or MG132. The proteins were separated on 12% SDS-PAGE gels and analyzed by Western blotting using a Myc antibody (1:1000). (**b**) Quantification of the relative levels of MycEhDUF2419. The data represent the mean ± SEM of three independent experiments, each with one to two technical replicates. Statistical significance is denoted by asterisks (* *p* < 0.05, ** *p* < 0.01, *** *p* < 0.001). (**c**) Left: Ponceau S stain showing the total protein labeling. Right: Western blotting was performed on the total protein extracts prepared from MycTrunEhDUF2419 overexpression trophozoites (24 μg/mL) treated with or without Q or MG132. The proteins were separated on 12% SDS-PAGE gels and analyzed by Western blotting using a Myc antibody (1:1000). (**d**) Quantification of the relative levels of MycTrunEhDUF2419. The data represent the mean ± SEM of three independent experiments, each with one to two technical replicates.

We initially expected that the addition of Q to the culture medium would result in elevated Q-tRNA^His^_GUG_ levels in trophozoites overexpressing MycEhDUF2419. However, contrary to our expectations, we observed a reduction in the Q-tRNA^His^_GUG_ levels following Q supplementation (Figure 7). This decrease, along with the identification of proteins associated with proteasome-mediated degradation pathways—such as RING-type E3 ubiquitin transferase (EHI_020100), E3 ubiquitin-protein ligase listerin (EHI_190430), and ubiquitin carboxyl-terminal hydrolase family protein (EHI_035180)—among those pulled down by the anti-c-Myc agarose bead immunoprecipitation suggests that the proteasome-mediated degradation machinery might regulate the levels of EhDUF2419 in the cell, subsequently affecting the Q-tRNA^His^_GUG_ levels. To validate this, we performed Western blot analysis with a Myc antibody on MycEhDUF2419 and MycTrunEhDUF2419-overexpressing trophozoites, treated with or without Q or the proteasome inhibitor MG132 [43]. The results showed that, in the absence of MG132 treatment, there was a significant reduction in the MycEhDUF2419 levels following Q treatment, while the reduction in the MycTrunEhDUF2419 levels with Q treatment was not significant (Figure 10). Following MG132 treatment, the MycEhDUF2419 levels significantly increased regardless of Q treatment, indicating that MycEhDUF2419 degradation is mediated by the proteasome (Figure 10a,b). This result explains that the significant reduction in the Q-modified tRNA^His^_GUG_ levels for MycEhDUF2419-overexpressing trophozoites after Q treatment compared to the control (Figure 7) is due to the decrease in the protein levels regulated by the proteasome. Although the MycTrunEhDUF2419 levels also showed an increasing trend with MG132 treatment (regardless of Q treatment), the change was not significant (Figure 10c,d).

## 4. Discussion

It has recently been established that DUF2419 salvages Q in many prokaryotes and eukaryotes [17], including *E. histolytica*, where direct salvage of Q from phagocytosed bacteria by the parasite has been demonstrated [16]. Structural analysis of *Sphaerobacter thermophilus* Qng1 (formerly DUF2419) has provided insights into the interaction of this enzyme with queuosine-5′-monophosphate, its primary substrate [17]. Despite this progress in understanding the DUF2419 biology, our knowledge of the proteins that interact with DUF2419 remains limited. STRING predicts possible interactions between EhDUF2419 and another nine proteins (Table S3). While these predictions offer valuable starting points for defining the DUF2419 interactome, they require experimental validation to confirm their relevance. In this study, we explored the interactome of EhDUF2419 to uncover additional functions of this protein. We identified 335 proteins that potentially interact directly or indirectly with EhDUF2419, including over 100 ribosomal proteins from both the 40S and 60S subunits, as well as numerous translation initiation factors, elongation factors, and aminoacyl-tRNA synthetases. The detection of EhDUF2419 in cellular fractions enriched with ribosomal proteins, along with the confirmed co-localization of MycEhDUF2419 and RPL1A, strongly supports the interactome data. This evidence suggests that EhDUF2419 is involved in translation through mechanisms that extend beyond its established role in salvaging queuosine, which is essential for the formation of Q-tRNAs involved in translation [9,10]. However, overexpressing EhDUF2419 as a Myc-tagged protein does not affect global protein synthesis, which may seem contradictory to this observation. Interestingly, it is well known that Q modification of tRNAs regulates specific processes, such as the translation of stress-related proteins or biofilm formation in bacteria, by influencing the translation of NAU codon-enriched genes [44]. Therefore, specific alterations in protein synthesis may have occurred, but they could not be detected using the SUnSET method.

The overexpression of EhDUF2419 in the parasite decreases the level of Q-tRNA^His^ in the presence of Q—a response not observed when a catalytically truncated version of EhDUF2419 is overexpressed. The mechanism behind this reduction in the Q-tRNA^His^ levels is unclear, but several scenarios are possible. The most likely explanation is that overexpression leads to improperly folded or non-functional enzyme forms acting as dominant negatives, or to the formation of non-functional aggregates that sequester the endogenous enzyme. This sequestration could reduce queuine processing, leading to decreased Q-tRNA^His^ formation. The validity of this scenario is supported by the observation that the proteasome inhibitor MG132 prevents the degradation of Myc-tagged EhDUF2419 regardless of Q’s presence in the culture medium, suggesting that misfolded or aggregated proteins are targeted for degradation. However, interactome analysis of EhDUF2419 detected only three proteasome-associated proteins, with no accumulation of heat shock proteins or other chaperones typically linked to misfolded proteins. This limited presence of misfolded or aggregated EhDUF2419 in the sample reduces the likelihood that the observed interactions reflect a non-specific response to unfolded proteins.

Among the proteins predicted by STRING to interact with EhDUF2419, only one, the PUA domain-containing protein (EHI_016460), has been identified in the EhDUF2419 interactome. PUA domain-containing proteins are involved in RNA modification and serve as translation factors [45]. This finding supports the notion that EhDUF2419 may interact with factors related to RNA processing and translation. However, further investigations are necessary to elucidate the precise nature of this interaction and its functional implications within the cellular context.

The possible interaction between EhDUF2419 and EhDNMT2 (Dnmt2) (EHI_103830) in *E. histolytica* revealed by the present interactome analysis could have significant implications for translation regulation. DNMT2, known for its role in 5-cytosine methylation (m5C) of tRNA, particularly near the wobble base, is crucial for the precise control of the translation efficiency [46,47,48]. This modification, which has been shown to enhance the translation efficiency in other organisms [48], might also affect the processing and function of tRNAs in *E. histolytica*. Previous studies indicate that Q modification of tRNA enhances the activity of DNMT2 homologs, such as Pmt1 from *Schizosaccharomyces pombe*, DnmA from *Dictyostelium discoideum* and EhDnmt2 in *E. histolytica* [9,11,49]. The dual modification of tRNAs by Q and m5C not only protects against endonucleolytic cleavage but also influences aminoacylation and codon–anticodon interactions [50]. Therefore, the interaction between EhDUF2419 and EhDNMT2 might modulate these processes through direct or indirect interaction, potentially impacting the translation of specific proteins and overall cellular function. Further investigation is needed to elucidate the exact mechanisms and consequences of this interaction within the parasite. It is also remarkable that other RNA modification enzymes were identified in the EhDUF2419 interactome, including RNA cytidine acetyltransferase (N-acetyltransferase 10, NAT10, EHI_033750), rRNA adenine N(6)-methyltransferase (EHI_013870), and RNA 3′-terminal phosphate cyclase (EHI_115370). These enzymes are involved in various aspects of RNA modification and processing, which could further elucidate the role of EhDUF2419 in regulating translation and RNA metabolism in *E. histolytica*.

Furthermore, the identification of interactions between various helicases and EhDUF2419 suggests a potential role for EhDUF2419 in regulating RNA metabolism. Helicases are essential for unwinding nucleic acids and are involved in multiple aspects of RNA metabolism. DEAD box helicases, in particular, play crucial roles in processes such as nuclear transcription, pre-mRNA splicing, ribosome biogenesis, nucleocytoplasmic transport, translation, RNA decay, and organellar gene expression [51,52]. Given that EhDUF2419 is a queuine salvage protein, its interactions with these helicases and other RNA-related proteins suggest an integrated role in modulating RNA metabolism and tRNA function. Queuine modification typically occurs within tRNA molecules, and helicases are known to interact with tRNAs, especially those with endonuclease-mediated “nicks” in their anticodon loops [53]. Additionally, RNA helicases can operate within acetyltransferase complexes that modify specific tRNA anticodons [54]. Therefore, the interaction between EhDUF2419 and helicases may influence the structure or folding of tRNA, potentially affecting its functionality and the associated translation processes. These findings suggest that EhDUF2419 may have a role in RNA metabolism beyond its involvement in queuine salvage, warranting further investigation to fully understand the implications of these interactions.

## 5. Conclusions

This study provides a comprehensive analysis of the interactome of EhDUF2419, highlighting its complex role in translation and RNA metabolism. Our findings reveal that EhDUF2419, a key player in queuine salvage, interacts with a diverse set of proteins involved in various aspects of translation and RNA processing. This includes interactions with ribosomal proteins, translation factors, helicases, and RNA modification enzymes. The evidence suggests that EhDUF2419′s function extends beyond queuine salvage, potentially influencing tRNA function and translation efficiency. The observed impact on the Q-tRNA^His^ levels further underscores its multifaceted role in the parasite’s cellular processes. These insights into the protein–protein interactions of EhDUF2419 pave the way for further research into its broader biological functions and regulatory mechanisms, offering new avenues for understanding its role in *E. histolytica*.

## Figures and Tables

**Table 1:** A list of the primers used for the construction of plasmids or qPCR.

Protein	Forward Primer (5′ to 3′)	Reverse Primer (5′ to 3′)	Enzyme Site	Notes
EhDUF2419	CCCCCGGGATGTGTGAATATGTTCG	CCCTCGAGTCAATAAAAAATGGTTTGTGTTCG	SmaI, XhoI	
TrunEhDUF2419	CCCCCGGGATGTGTGAATATGTTCG	CCCTCGAGTCAGCGATATCCTTCAATAAAT	SmaI, XhoI	
EhDUF2419	TCCATCTGGGTCTGAAGAAG	GTTTGTGTTCGGTGGTGTGG		qPCR
rDNA	TCAAAAAGCAACGTCGCTA	AGCCCGTAAGGTGATTTCT		qPCR

## Data Availability

Data are contained within the article and Supplementary Materials.

## Supplementary Materials

The following supporting information can be downloaded at https://www.mdpi.com/article/10.3390/cells13221900/s1, Figure S1: Comparative analysis of the sequence alignment and structural diagrams of the EhDUF2419 and StDUF2419 proteins. Figure S2: Protein synthesis level of the control, MycEhDUF2419 and MycTrunEhDUF2419 overexpressed trophozoites with or without Q treatment using the SUnSET method. Table S1: List of all the proteins interacting with EhDUF2419 that were enriched by IP in three independent experiments. Table S2: STRING enrichment and cluster analysis of 335 protein interactions. Table S3: STRING analysis of EhDUF2419 interaction. Table S4: Description of the parameters that are given in Table S1.
