# Supplementary material for: Exploring the Interactome of the Queuine Salvage Protein DUF2419 in Entamoeba histolytica
Source: Cells. 2024 Nov 18;13(22):1900. doi: 10.3390/cells13221900 (PMC11592518; doi:10.3390/cells13221900)
Supplement: Supplementary file 1 [file cells-13-01900-s001.zip › Figure S1 Comparative analysis of sequence alignment and structural diagrams of EhDUF2419 and StDUF2419 proteins. .pdf]

```
Identities:93/285(33%), Positives:149/285(52%), Gaps:18/285(6%)

StDUF2419  42    QVTPPTWNRELHWS-DGREALANYILVLDAVNFCFWGEPRWRIEYAGAVYDGYWALAASL  100
                 ++   T++  +H + +  E+   YILV+DA+NFCFW          G  YD    L   L
EhDUF2419  36    KIEAETFDESIHQAPNDIESRLRYILVVDALNFCFWPT-------EGFEYDD---LTKGL  85

StDUF2419  101   KRALEQGVPLTDASYLAEITRDDVATIFAGEGE-IPLLDERARILRETGSVLAERFAGRF  159
                  R       + + + +  ++   ++         I  ++ER R++RE G VL  RF  +
EhDUF2419  86    SRLEHDHPEVFEPNQMKHVSSCLLSQYLVYNNRVISNIEERTRLMREVGEVLCNRFDQKV  145

StDUF2419  160   SDAIAAAGRSAVALVDIVTNAFPSFRDVATYRGEQVRFYKRAQILVSDLYGAFDGSDLGA  219
                  + +  +   A  LV ++   FP FRD   Y+G QV FYKRAQI+VSD+ G       G
EhDUF2419  146   LNLLEESKYDATTLVSLIAKEFPGFRDSTIYKGRQVFFYKRAQIVVSDIQGM-----CGC  200

                            * *
StDUF2419  220   FDDLGELTAFADYKVPQVLHHLGILRYAPALHDRLARREEIPAGSPEEVEIRAATIWGVE  279
                    L +LT FADY++PQVL    +L     L  ++  ++EIP+GS EE+EIR   +  ++
EhDUF2419  201   IKGLEQLTGFADYRIPQVLLGWDVLDIEDQLKQKILDKKEIPSGSEEEIEIRCTVLSAIK  260

                                    *   *
StDUF2419  280   ELRRA-LASRGHALDAYQVDWLLWDEGQRLPAGTLPYHRTRTIFY  323
                  ++   L ++   ++ Y++DW LW  G++      P+HRT+TIFY
EhDUF2419  261   MIQAIFLETKNSFIEGYRIDWFLWSYGEKNKEQLPPHHRTQTIFY  305
```

(**a**)

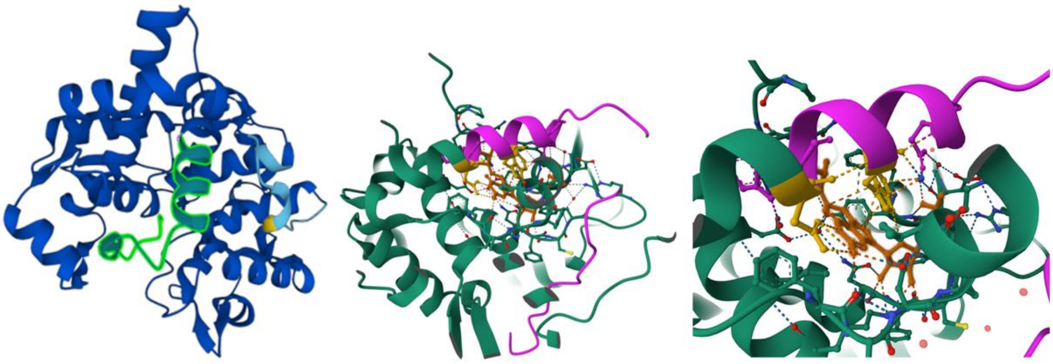

(**b**) (**c**) (**d**)

Figure S1: Comparative analysis of sequence alignment and structural diagrams of EhDUF2419 and StDUF2419 proteins. (**a**) Sequence alignment shows that EhDUF2419 shares 33% identity with StDUF2419, with active site residues marked as pink stars in StDUF2419. The middle line indicates the quality of the match: identical matches are shown as letters, positive matches (conservative substitutions) are indicated by a + sign, and white spaces denote matches with zero or negative scores. Dashes represent gaps in the sequences. (**b**) Predicted AlphaFold structure of EhDUF2419, with the green section representing the truncated part. (**c, d**) Full or magnified view of the crystal structure of StDUF2419 in complex with queuosine-5'-monophosphate (PBD: 7U91). The orange section represents the ligand queuosine-5'-monophosphate, the magenta section corresponds to the truncated part, and the yellow sections highlight the active site residues D298 and W302, indicating these residues directly interact with the ligand.
