# Supplementary material for: Exploring the Interactome of the Queuine Salvage Protein DUF2419 in Entamoeba histolytica
Source: Cells. 2024 Nov 18;13(22):1900. doi: 10.3390/cells13221900 (PMC11592518; doi:10.3390/cells13221900)
Supplement: Supplementary file 1 [file cells-13-01900-s001.zip › Figure S2 Protein synthesis level of empty vector, MycEhDUF2419 and TrunEhDUF2419 overexpressed trophozoites with or without Q treatment using the SUnSET method.pdf]

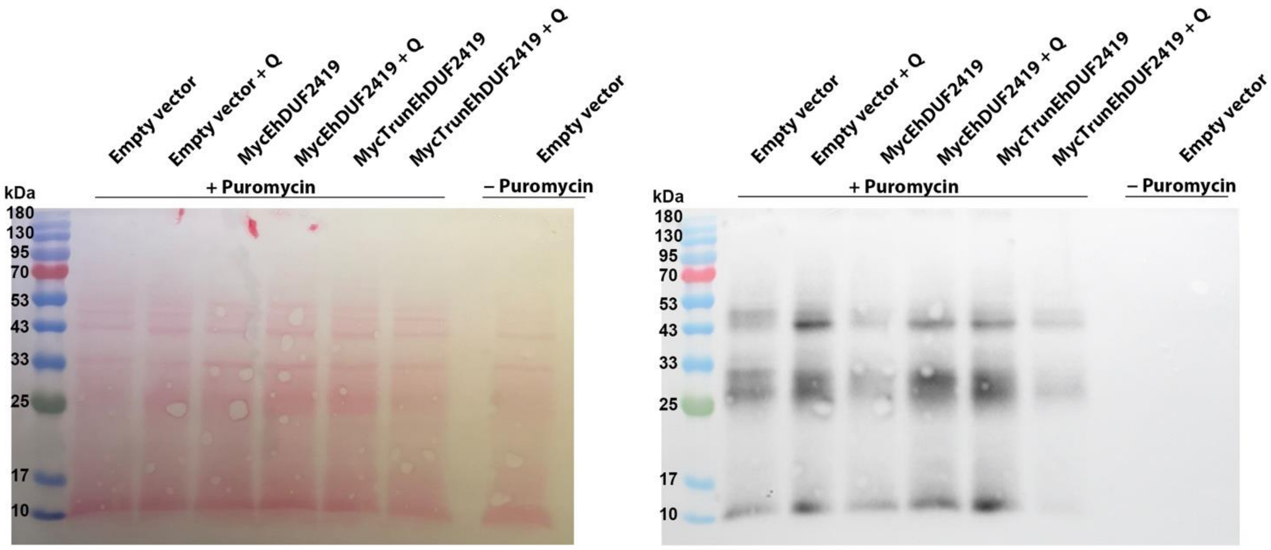

(a)

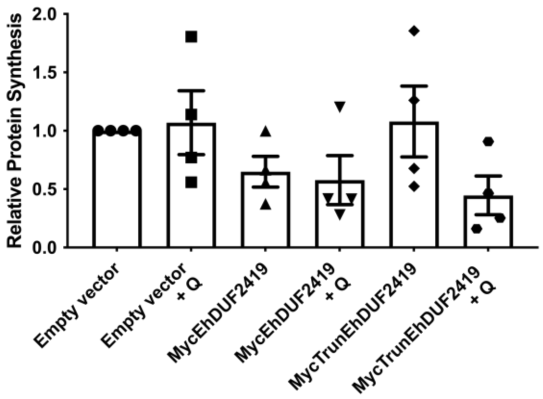

(b)

Figure S2. Protein synthesis level of empty vector, MycEhDUF2419 and MycTrunEhDUF2419 overexpressed trophozoites with or without Q treatment using the SUnSET method. (**a**) Left: ponceau S stain showing total protein labeling. Right: Western blot analysis using a puromycin antibody to detect puromycin-labeled nascent polypeptides. Trophozoites were labeled with puromycin at a concentration of 10 µg/ml. Additionally, trophozoites were pre-incubated with Q (0.1 µM for 2 days) prior to puromycin labeling. (**b**) Densitometry analysis was conducted to quantify the fold change in protein synthesis across different trophozoite conditions with or without Q, normalized to the empty vector control. Data are presented as means ± SEM from four independent experiments. No significant differences were observed.
